# Specialized Cortex Glial Cells Accumulate Lipid Droplets in *Drosophila melanogaster*

**DOI:** 10.1371/journal.pone.0131250

**Published:** 2015-07-06

**Authors:** Viktor Kis, Benjámin Barti, Mónika Lippai, Miklós Sass

**Affiliations:** Department of Anatomy, Cell and Developmental Biology, Eötvös Loránd University, Budapest, Hungary; University of Houston, UNITED STATES

## Abstract

Lipid droplets (LDs) are common organelles of the majority of eukaryotic cell types. Their biological significance has been extensively studied in mammalian liver cells and white adipose tissue. Although the central nervous system contains the highest relative amount and the largest number of different lipid species, neither the spatial nor the temporal distribution of LDs has been described. In this study, we used the brain of the fruitfly, *Drosophila melanogaster*, to investigate the neuroanatomy of LDs. We demonstrated that LDs are exclusively localised in glial cells but not in neurons in the larval nervous system. We showed that the brain’s LD pool, rather than being constant, changes dynamically during development and reaches its highest value at the beginning of metamorphosis. LDs are particularly enriched in cortex glial cells located close to the brain surface. These specialized superficial cortex glial cells contain the highest amount of LDs among glial cell types and encapsulate neuroblasts and their daughter cells. Superficial cortex glial cells, combined with subperineurial glial cells, express the Drosophila fatty acid binding protein (Dfabp), as we have demonstrated through light- and electron microscopic immunocytochemistry. To the best of our best knowledge this is the first study that describes LD neuroanatomy in the Drosophila larval brain.

## Introduction

Lipid droplets (LDs) are common organelles of the majority of eukaryotic cell types. These structures are generally regarded as the cellular storage sites of lipids, including neutral lipids (sterols, triacylglycerols) and a repository for the precursors of phospholipids, the building blocks of cellular membranes [1,2]. During the last decades considerable data has emerged in the literature proving that LDs serve not merely as passive stores of excess fat and other lipid substances, but they simultaneously participate in many different cellular processes, such as intracellular protein and phospholipid metabolism during cell division [3], the replication of the Hepatitis C virus [4, 5], and proteasomal protein degradation [6]. A hallmark of LD research was the discovery that LDs function as a protein storage depot in cells. The presence of over 150 various proteins has been linked to LDs [7, 8]. Moreover, LDs are involved in intracellular protein metabolism [5,7]. There is ample evidence proving that LDs play a crucial role in the pathophysiology of certain human diseases, such as obesity, metabolic syndrome, fatty liver syndrome and atherosclerosis [9–13]. LD cell biology and physiology have been extensively studied in liver cells and in the adipose tissue of mammals.[14–16] Although the nervous system contains the highest relative amount of lipids, the spatiotemporal distribution and physiological function of LDs in the brain remain largely unknown. The *Drosophila melanogaster* is an excellent genetic model for higher animals, since in contrast with mammalian systems, gene redundancy is minimal in flies, allowing scientists to analyze *in vivo* gene functions. The Drosophila also has a short life cycle, a wide variety of available genetic tools, and mutants and RNAi lines have been systematically generated [17]. The most powerful genetic tool in Drosophila is the Gal4-UAS dual transgenic system, where Gal4 is a transcription factor that selectively binds to the Upstream Activating Sequences (UAS) and enhances the expression of the downstream DNA sequences [18]. This allows a variety of transgenic techniques such as targeted gene expression modification (overexpression or RNA silencing) by expressing the Gal4 under the control of tissue-specific promoters and fusing transgenes or ds RNA sequences after the UAS. Moreover, while a large portion of the *Drosophila* neurodegeneration mutants (*bubblegum*, *swiss cheese*, *loechrig*, *ApoD*, *frataxin*, *sicily*) [19–23] affect lipid metabolism and disturb LD homeostasis, neither the cellular, nor the spatio-temporal distribution of LDs has been described to date in *Drosophila*. In this paper, we used the brain of the fruitfly to study lipid droplet anatomy in the larval nervous system.

## Methods

### Reagents

Unless otherwise indicated, all reagents and materials used for this study were obtained from Sigma-Aldrich (St. Louis, MO).

### Fruitfly stocks and genetics

Flies were raised on standard yeast/cornmeal/agar media, at 25°C, 50% humidity and a 12-hour light/12-hour dark daily cycle, under uncrowded conditions. For the RNAi experiments, flies were raised at 29°C to reach the maximum efficacy of the apllied Gal4-UAS directed expression system. The following *D*. *melanogaster* stocks were used: Oregon R, Nrv2-GFP (BDSC stock no. 6828), repoGal4 (BDSC, stock no. 7415), UAS-CD2-HRP (BDSC, stock no. 9906), UAS-Dfabp RNAi (Transgenic RNAi Project—HMS01163), UAS-myr-RFP (BDSC, stock no. 7119), repoflp (gift from Christian Klämbt, Institut für Neurobiologie, Universitat Münster, Münster, Germany); UAS-Lsd2-EGFP (gift from Ronald P. Kühnlein, Max-Planck-Institut für Biophysikalische Chemie, Göttingen, Germany), cortex glia specific Gal4 driver (NP2222, Kyoto Stock Center), Act > CD2 > GAL4 (gift from Gábor Juhász, Eötvös Loránd University, Budapest, Hungary), *dfabp* ^*EP3252*^ (Szeged Stock Center), Dfabp-GFP (115–074, Kyoto Stock Center). Oregon R flies were used as control for the histological experiment. For the RNAi experiments, control animals carried the same chromosome set except for the UAS-dfabp-RNAi transgene containing chromosome which was replaced with a wild type one (Oregon R).

### Generation of flip-out clones

The following genotypes were generated through multiple crossing steps: repoFlp/+; UAS-Lsd2-EGFP, UAS-myr-RFP/ Act > CD2 > GAL4 for the analysis of glial cell morphology and the LD profile. repoFlp/Nrv2-GFP; UAS-myr-RFP/ Act > CD2 > GAL4 for validating the identity of lipid droplet accumulating superficial cortex glial cells. Flies with these genotypes due to the low efficacy of the Flp recombinase contained a very few myr-RFP-labeled single glial cells.

### Production of the Dfabp antisera

Molecular cloning techniques were performed according to standard procedures. PCR amplification of the third Dfabp (CG6783) exon was done using ExTaq DNA polymerase (Takara) with the primers 5’ GGATCCGGCGTCGGTCTGGTGACGCG 3’ and 5’ CTGCAGGGTGATCAGCTCGTTGTCGG 3’. The PCR product was directly cloned into the pQE-UA bacterial expression vector (Qiagen), the recombinant protein encoding the partial Dfabp protein, fused to an N-terminal hexahistidine tag was expressed in *E*. *coli* M15 cells. Protein purification was performed using the QIAexpressionist kit of Qiagen. Mice were immunized with the fusion protein, and the resulting polyclonal antisera (internal code: 3A1) were used for further investigation.

### Western blotting

20 mg of mutant and control larvae was washed twice with PBS and was homogenized in 40 μl of proteinase inhibitor cocktail (Roche) dissolved in PBS. Equal volume of standard Laemmli’s buffer was added. The homogenate was boiled immediately for 5 minutes, pelleted at 10000g for 10 minutes at room temperature (RT) and the middle fraction was collected. Protein samples were separated on 12% polyacrylamide gel and were transferred to nitrocellulose membrane (Bio-Rad). After incubation in blocking solution (3% milk powder in 0,05% Tween-20/TBS, hereafter TBST) for 1 hour at RT, membranes were incubated with primary antibody (1:5000) in antibody solution (1% milk in TBST) overnight at 4°C, followed by three 10-min washes in TBST. Signals were detected using alkaline phosphatase-coupled secondary antibodies, diluted 1:3000 in antibody solution. Finally, membranes were developed by freshly prepared BCIP/NBT solution (Bio-Rad).

### Histology, immunostainings and imaging

For immunostainings, brains were fixed in 4% formaldehyde (freshly depolymerized from paraformaldehyde) in PBS for 30–60 min. After several washes, free aldehydes were reacted with 50-50mM ammonium chloride—glycine dissolved in PBS. Samples were permeabilized with 0,1–0,15% Triton X-100- PBS (hereafter PBTx) and blocked in 20% FCS for 30 min. Samples were incubated for two days at 4°C with the following concentrations of primary antibodies; anti-Dfabp 1:1000, anti-Repo 1:20 (DSHB), anti-GFP 1:1000 (Abcam, cat no. ab290-50). After several washes in PBTx, brains were incubated with the apropriate secondary antibodies diluted 1:800 in PBTx: Alexa568-coupled goat anti-mouse (Invitrogen), Alexa488-coupled goat anti-mouse (Invitrogen), FITC-coupled goat anti-rabbit. After the incubation with the secondary antibodies brains were extensively washed (4x30 min in PBTx). The first washing solution contained 1μg/ml DAPI to stain nuclei. Finally samples were mounted with Vectashield (Vector) and stored at 4°C in the dark. Slides were analyzed with a Zeiss Axiomiager Z1 fluorescent microscope equipped with an Apotome grid confocal unit using Mrm1 camera and AxioVision 4.82 imaging software. For the Dfabp-Repo double immunostaining, since both Dfabp and Repo antisera were raised in mouse, a Dfabp-GFP expressing Drosophila protein trap line (115–074, Kyoto Stock Center) and anti-GFP instead of anti-Dfabp antibody was used. The Dfabp-GFP signal was completely identical with the Dfabp immunostaining. For the imaging of animals with single labeled cells or carrying reporter constructs, larvae were dissected, stored (for up to 20 minutes) and mounted in icecold PBS then photographed immediately in a fluorescent microscope using ApoTome grid confocal unit.

### Oil Red O staining

Animals were dissected in icecold PBS and fixed in a solution containing: 2% formaldehyde, 0.5% glutaraldehyde, 3mM CaCl_2_ and 1% sucrose in 0.1M Na-cacodylate, pH 7.4 (60 min, RT). Samples were rinsed twice in distilled water and stained for 30 min in Oil Red O staining solution (freshly prepared from 6ml of 0,1% Oil Red O in isopropanol and 4 ml distilled water). Samples were then mounted in pure glycerol and stored at RT.

### Semithin sections

Semithin (0,5 μm) sections from Durcupan embedded blocks were cut with glass knives stained in 1% toluidine blue– 1% azure II solution mounted in pure glycerol and viewed in a Zeiss AxioImager Z1 microscope with a 63x oil immersion objective, using an ICc1 camera. For all light microscopic images white balance was adjusted on empty resin.

### Routine electron microscopy and HRP cytochemistry

Animals were dissected in icecold PBS and brains were fixed in a solution containing: 2% formaldehyde, 0.5% glutaraldehyde, 3mM CaCl_2_ and 1% sucrose dissolved in 0.1M Na-cacodylate, pH 7.4 (60 min, RT), postfixed in 0,5% osmium tetroxide (60 min, RT) and 1% aqueous uranyl acetate (30 min RT), dehydrated, embedded in Durcupan and cured for 60 hours at 60°C. Ultrathin (80nm) sections were stained with lead citrate for 3 min. Grids were analyzed in JEOL JEM 1011 transmission electron microscope operating at 60kV. Images were taken with an Olympus Morada 11 megapixel camera and iTEM software (Olympus). For HRP cytochemistry, brains were fixed in the same way as described above except that the fixative did not contain formaldehyde, since it strongly reduces the activity of HRP. Endogenous peroxidase-like activity was quenched in 1% hydrogen peroxide for 10 min. After several washes, samples were preincubated in standard DAB solution without hydrogen peroxide for 30–60 min, then developed in DAB solution supplemented with 0,01% hydrogen peroxide for 30min. Brains were then postfixed in 0.5% osmium tetroxide for 45 min. En bloc staining with uranyl acetate was ommited. Samples dehyrdated and embedded in Durcupan as described above.

### Freeze substitution and LR White embedding

For low temperature embedding brains were fixed in a solution containing: 4% formaldehyde (freshly depolimerized from paraformaldehyde), 0,05% glutaraldehyde and 0,2% picric acid dissolved in 0,1M PB pH 7,4 for 60min at RT. After extensive washing, free aldehyde groups were quenched as described above and samples were cryoprotected in successive incubations in 10%, 20%, 30% buffered solutions of sucrose for 4, 12 and 24 hours respectively. Then samples were freezed in liquid nitrogen and transferred into anhydrous methanol for 24 hours at -20°C. All the following steps were done at -20°C. Samples were infiltrated for 24 hours with pure LR White containing 2% benzoyl peroxide as a catalysator. Curing was done using a home made UV chamber with two DL-103 2x6W UV lamp for 48h at -20°C.

### Post-embedding silver-intensified immunogold labeling of freeze-substituted LR White-embedded sections

Ultrathin (80-90nm) sections cut from low temperature-embedded LR White blocks were collected on formvar-coated 100 mesh nickel grids. All the immune reactions were carried out in humidified Parafilm-coated 96 well plates. Steps of the procedure were the following:

(1) 5% H_2_O_2_ for 1 min; (2) biDW for 3 x 5 min; (3) 10% FCS in 50mM glycin dissolved in TBS pH 7,6 for 30 min; (4) anti-Dfabp antibody (1:300) in 5% FCS-TBS overnight at 4°C; (5) 3 x 5 min 2% FCS-TBS, (6) 10nm gold-conjugated goat anti-mouse secondary antibody diluted 1:100 in TBS-2%BSA for 90 min, RT; (7) 3 x 5 min TBS; (8) 1% glutaraldehyde in TBS for 10 min; (9) extensive washing in biDW; (10) silver intensification of 10 nm gold particles for 8 min (R-GENT SE-LM, AURION); (11) extensive washing in biDW; (12) staining grids with uranyl acetate for 15 min and with lead citrate for 2 min; (13) air drying.

### Quantification and data anaylsis

Images were analysed with AxioVision 4.82 (for LM images) and iTEM (for EM images) software. Primary images were edited using Adobe Photoshop CS5 software: area of interest was cropped, brightness and white balance were adjusted where necessary. LDs were counted manually. During embedding, we paid outmost care on the orientation of the specimens, and before cutting ultrathin sections, we observed semithin sections to make sure that the medial part of the central brain is in the cutting surface. This slow procedure resulted in a highly reliable dataset which was a prerequisite for further analysis. For every measurement, data were weighted for the tissue area to exclude unwanted biases. Tissue area was measured using AxioVision 4.82 software (for LM images) and iTEM (for EM images). This way, we generated the LD per tissue area ratio data. Images for the stastical analysis (10–13 per each celly types) were taken from 5 animals. Data were summarized in Excel (Microsoft). Statistical analysis was performed using IBM SPSS Statistics software applying the indicated tests, and box plot figures were generated using the same software. On box plots, bars show the data, lying between the upper and lower quartiles, median is indicated as a horizontal line within the box. Whiskers plot the smallest and largest observations while dots and asterisks indicate outliers. P<0.05 was considered to be significant. * means p<0.05, ** means p<0.01, *** means p<0.001.

## Results

### LDs present in the Drosophila brain

**Fig 1:**
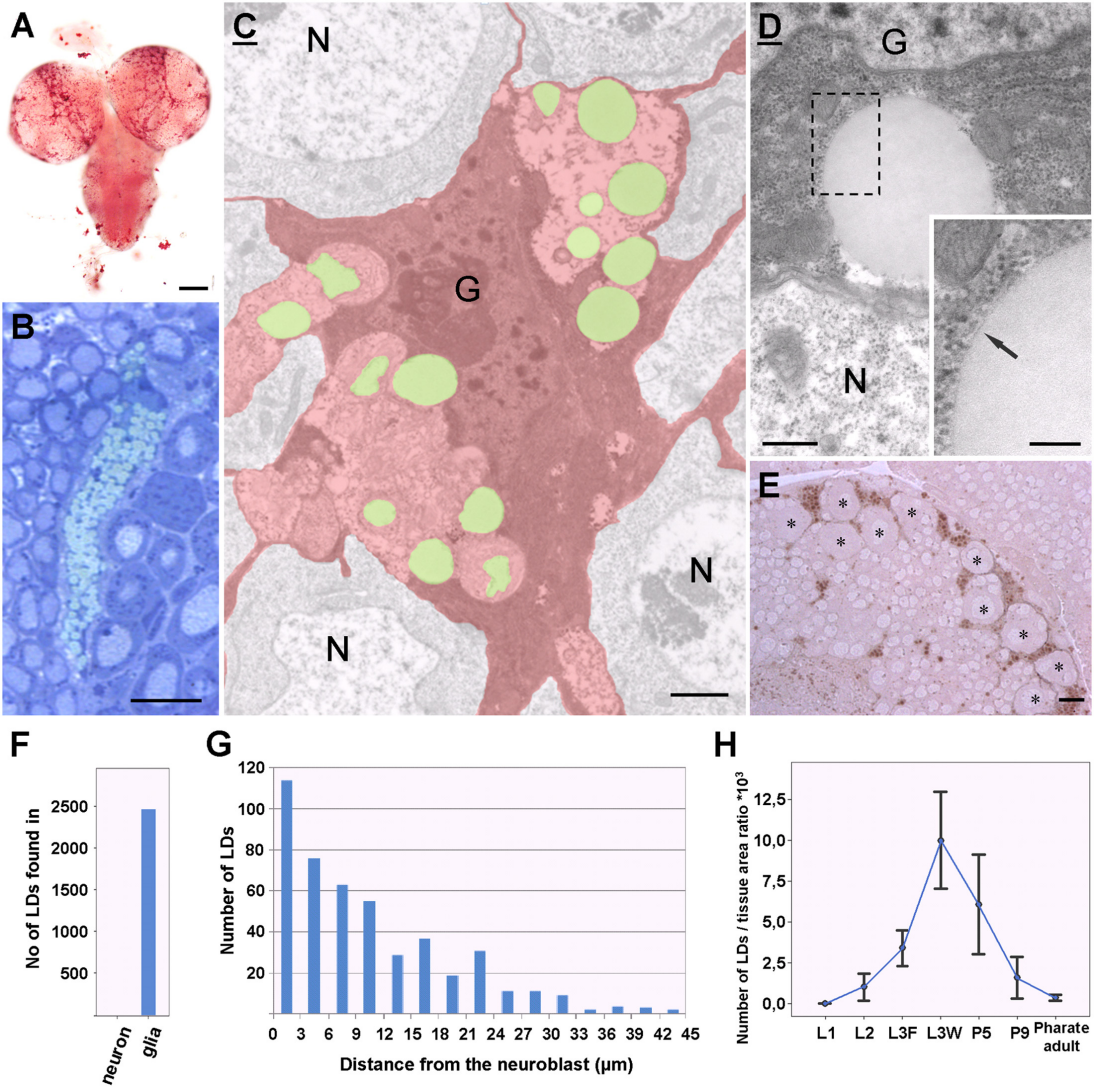
Lipid droplets in the *Drosophila* brain. (A-E) Pictures taken from third instar larval brains. (A) Oil Red O staining, note an intense staining in the dorso-medial part of the central brain. (B) Toluidine blue-stained semithin section from the brain cortex. LDs (green) are organized in large clusters between neurons. (C) Pseudocolored electron micrograph taken from the brain cortex. LDs (green) are found in the cytoplasm of glial cells (G, red) but not in neurons (N). (D) High power electron micrograph from the perinuclear region of a cortex glia (G) LDs are rounded electron-opaque structures, delineated by a phospholipid monolayer (inset, arrow). N: neuron. (E) LDs (brown) are generally found in the closest vicinity of neuroblasts (asterisks). Unstained semithin section. (F) Diagram representing the distribution of LDs between neurons and glial cells. (G) Number of LDs in order of their distance from neuroblasts. (H) Time-course changes in the amount of accumulated LDs per brain tissue area ratio. Standard deviations are indicated. Scalebars: (A) 100 μm (B) 10 μm (C) 1μm, (D) 500nm, inset: 200nm, (E) 10 μm.

To visualize the lipid droplets, we stained third instar larval brains with Oil Red O, a traditional neutral lipid dye. LDs were present throughout the entire brain but were preferentially enriched in the medial part of the central brain (Fig 1A). As the density of LDs was highest in this brain area, our research focused on this particular region. We found LDs to be organized in large clusters in resin sections stained with toluidine blue, which stains LDs green in a metachromatic manner (Fig 1B). In the electron microscope (EM), LDs were found exclusively in the glial cells, which could be identified on the basis of their heterochromatic nucleus and dense cytoplasm packed with ribosomes [24] (Fig 1C). To validate the glial nature of the lipid accumulating cells in the brain, we labeled glial membranes with membrane targeted HRP (S1 Fig). The LDs found in *Drosophila* glial cells shared the properties of LDs observed in mammals: homogenous electron-opaque staining and delineation by a phospholipid monolayer [25] (Fig 1D) The LDs were concentrated mostly in the perinuclear region of glial cells (Fig 1C and 1D). Remarkably, no LDs were found in neurons (2483 LDs counted from 15 brains, Fig 1F).

Next, we investigated the overall distribution of LDs in the medial part of the central brain. Interestingly, we determined that 65.5% of LDs (310 of 473 LDs) were found in the closest vicinity (<10 μm) of neuroblasts, located in the outer layers of the brain cortex (Fig 1E and 1G). Neuroblasts are large (>10μm in diameter) neural precursors located beneath the surface of the brain [26]. These cells can be identified on the basis of their size and their euchromatic nucleus. Usually large peptiderg neurons are in the same size, but neuroblasts lack neural processes, dense neurosecretory vesicles and extended Golgi-apparatus. Next, we wanted to quantify the changes in the number of accumulated LDs in the course of development. We found that while LDs are absent in the embryonic stage and in the first instar larvae, the density of LDs gradually increases from the beginning of the second larval stage until the end of the wandering period of the last larval stage, when it reaches its highest value, after which it decreases gradually until the end of the pupal stage (Fig 1H). Again, virtually no LD is found in the brain of a pharate adult fly. This dataset suggests that the brain’s LD pool is not constant but actually changes dynamically during development.

### LDs are accumulated in specialized cortex glial cells

**Fig 2:**
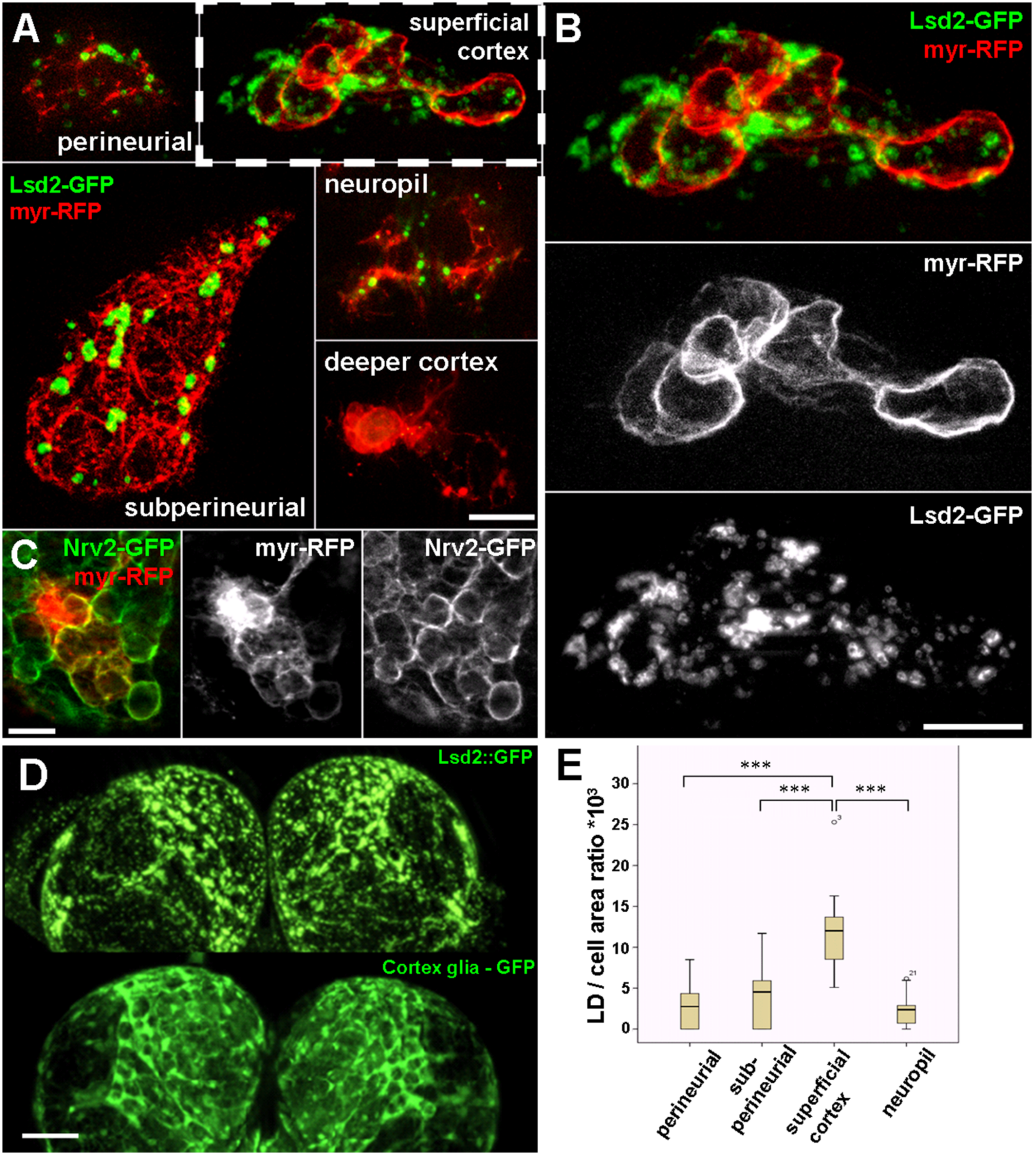
Cell type-specific distribution of LDs. (A) Single cell clones of different glial cell types. Glial cell membranes are higlighted by myr-RFP (red) and LDs are highlighted by Lsd2-GFP (green). (B) Higher magnification image of a superficial cortex glia shown in panel A. (C) A superficial cortex glial clone (myr-RFP, red) on a pan-cortex glial cell labeled background (Nrv2-GFP, green). Note the similar morphology of the labeled glia as shown in panel B. (D) Pattern of LDs in the larval brain, visualized by ectopically expressed Lsd2-GFP shows similar distribution as the cortex glial specific GFP signal. (E) Box plot representing LD per cell area ratios from particular glial cell types. Scalebar: (A,B,C) 20 μm (D) 100 μm

**Fig 3:**
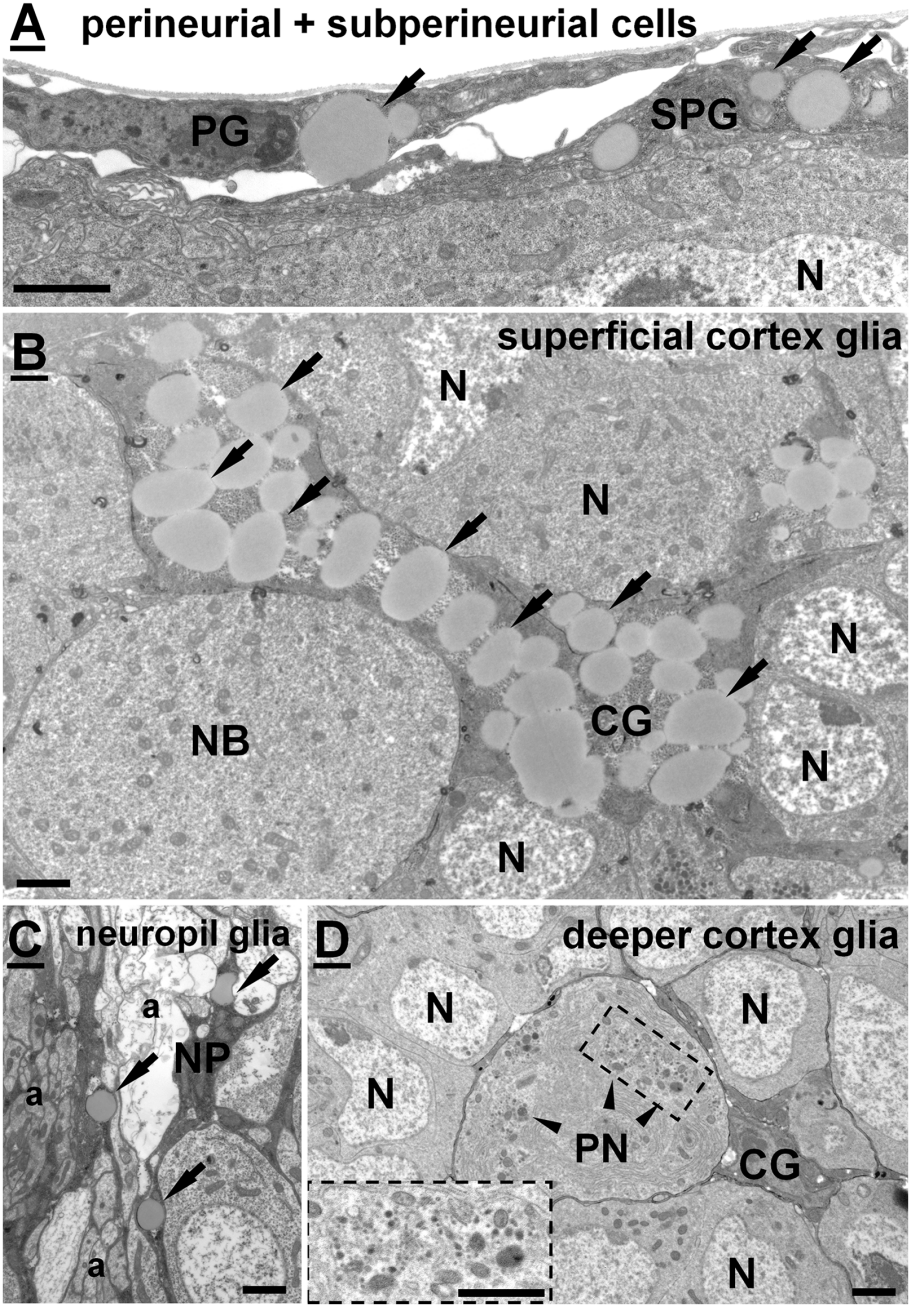
Representative electron micrographs of particular glial cell types. LDs are marked by arrows. (A) Perineurial (PG) and subperineurial cells (SPG) located on the surface of the brain. (B) A superficial glial cell located in the outer layer of the brain cortex, containing high amount of LDs. (C) A neuropil glia (NP) located at the cortex-neuropil boundary ensheating axons. (D) A deeper cortex glia (CG) found close to the cortex-neuropil boundary encapsulating a large peptiderg neuron (PN) and several other neurons (N) with its processes. No LDs seen in the cytoplasm of such a cortex glia. Note the presence of large clusters of neurosecretory vesicles (arrowheads) in the cytoplasm of PN. Scalebar: 2μm.

Since we found LDs exclusively in glial cells, we sought to determine whether LDs are distributed equally between glial cell types, or if each cell type has a different LD content. To answer this question, we generated genetically labelled single glial cells in which glial membranes were highlighted with myr-RFP and LD content was labeled with Lsd2-GFP (Fig 2A). Lsd2 (Lipid storage droplet 2) is the *Drosophila* orthologue of the mammalian perilipin2, a widely used marker of LDs [27, 28]. This double labeling technique allowed us to identify glial subtypes through their morphology, and to determine their LD content simultaneously in the same cell. Drosophila larval glial cells can be classified into four types: perineurial, subperineurial, cortex and neuropil glia [29]. The smallest are perineurial cells (PG,), filopodial shaped glial cells located at the surface of the brain. Subperineurial cells (SPG) are very large, flat shaped, polyploid cells (>150μm in width) located just below the perineurial layer. SPGs are connected to each other through septate junctions, through which they form the blood-brain barrier in the *Drosophila* brain [30,31]. Cortex glial cells (CGs) are multipolar, form a delicate network in the cortex of the brain, and insulate neuronal cell bodies with very thin processes. Neuropil glia (NP) has two subclasses: the ensheathing glia which has flattened cell bodies and lacks processes penetrating into the neuropil; and the reticular glia, which has many processes that invade the neuropil and encapsulate axon terminals and dendrites [20,24]. NP cell bodies can be located at the cortex-neuropil boundary or in the neuropil. Each glia type described above contained different amounts of LDs during the clonal analysis but the cortex glial cells located in the superficial region of the brain cortex accumulated significantly more LDs in comparison to the other types (Fig 2A and 2B). These lipid accumulating cortex glial cells had characteristic processes encapsulating unlabeled large areas, possibly cell bodies of neurons. Based on the location and the size of these unlabeled profiles, we propose that they could be neuroblasts, known to be located close to the brain surface. Interestingly, we found some cortex glial cells that contained no LDs (Fig 2A, Fig 3D). These cells had very thin filopodial processes and were located in the deeper regions of the brain cortex (S2 Fig). To validate our results obtained with single cell labeling, we also generated myr-RFP labeled clone cells on a genetic background where all the cortex glial membranes were highlighted with a GFP tagged version of the glial specific Na^+^,K^+^-ATPase—Nrv2 [32]. The morphology of cortex glial clones labeled this way was identical to the lipid accumulating cells observed in the superficial cortex during LD analysis (Fig 2C). We also compared the distribution of LDs with the pattern of specific glial subtypes and found that the signal of fluorescently labelled LDs (Lsd2-GFP expressed in glial cells) strongly overlapped with the pattern of cortex glia in whole mount larval brains (Fig 2D). We quantified this observation on EM sections where glial cell types can be identified through their soma location, the morphology of their processes, and the neural elements to which they are attached (Fig 2E). We found that cortex glial cells located close to the brain surface possessed the highest LD number/cell area ratio (11,8±4,6), while other glial types contained notably less LDs: PG (2,6±2,5), SPG (4,1±3,5), NP (2,1±1,7). For the represesentative EM images of each glial type listed above, see Fig 3. In the EM, we also found cells in the deeper cortex insulating neuronal cell bodies, containing no LDs (Fig 3D). These cells may represent a specific subclass of cortex glial cells. These data suggest that (superficial) cortex glial cells are the main storage depositories of LDs in the larval brain.

**Fig 4:**
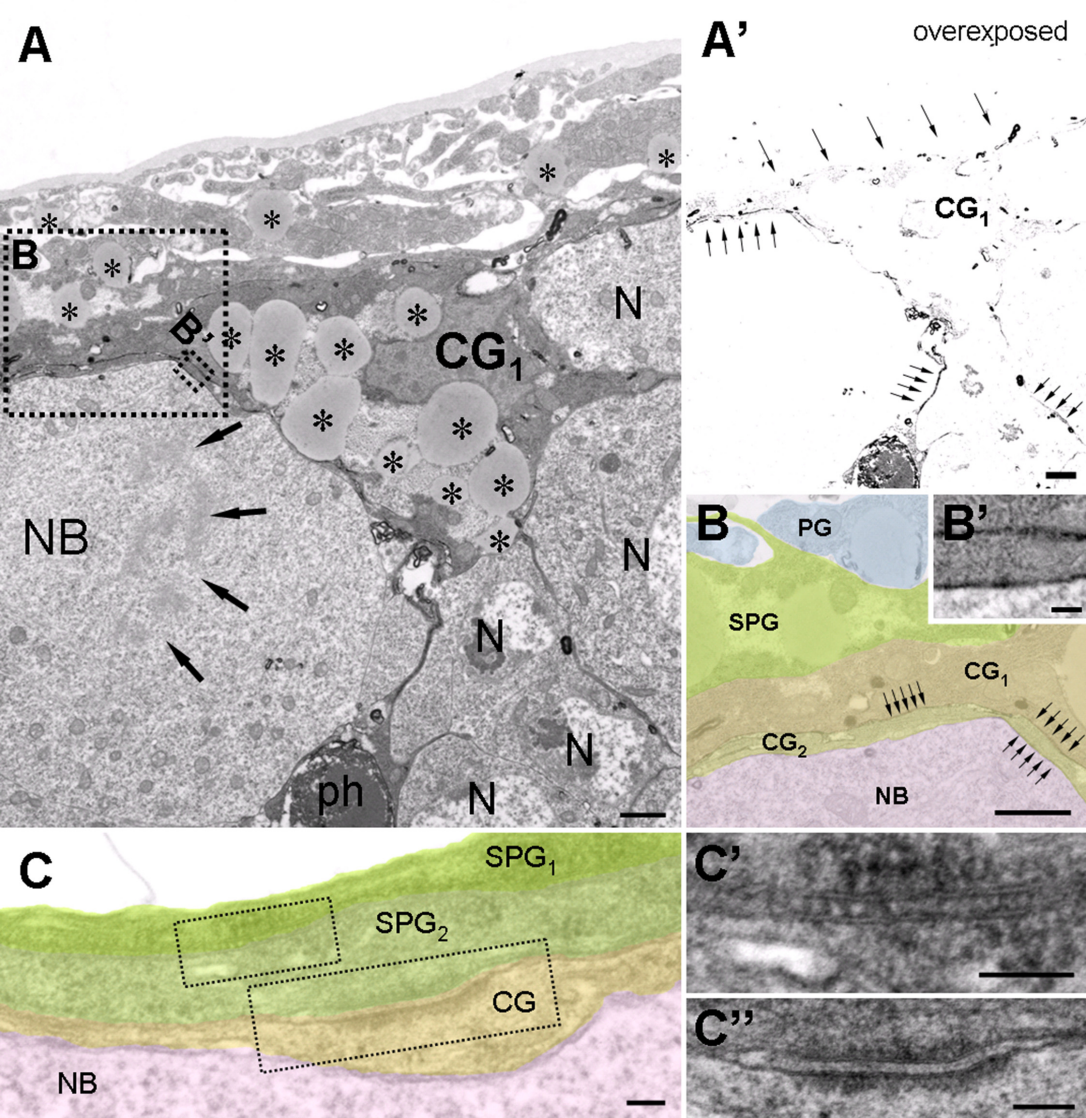
Ultrastructural features of the LD accumulating superficial cortical glial cells. (A) HRP-labeled superficial cortical glial cell (CG_1_), encapsulating a mitotic neuroblast (NB, arrows: chromosomes) and other neurons (N). Note the abundance of LDs. (A’) Same picture as shown in panel A but overexposed during acquisition to reveal the DAB stained processes (arrows) at low magnification. Note that a phagosome (ph, labeled in panel A) accumulates HRP. (B, B’) Higher magnification pseudocolored image taken from the dashed areas in panel A. DAB stained glial membranes are indicated (arrows). Two overlapping cortex glial processes (CG1 and CG2) insulate a mitotic neuroblast (NB). (C) Organization of the glial layers encapsulating neuroblasts. (C’) Subperineurial glial cells (SPG_1-2_) are connected to each other through septate junctions. (C”) A subperineurial (SPG_2_) and a superficial cortex glia (CG) are connected with an adherens junction. Scalebar: (A, A’, B) 1 μm, (B’,C, C’, C”) 100nm.

To verify our observations at the EM level, we labeled cortex glial membranes with a membrane-targeted HRP, which can be visualised after the DAB reaction [33]. As expected, we found that these superficial cortex glial cells insulated mitotic neuroblasts (Fig 4A). The cells accumulated high amounts of LDs in their perinuclear region, had invaginated dense heterochromatic nucleus and often also contained a high amount of glycogen. The cells were tightly attached to subperineurial cells. Another interesting finding that EM showed was that while neighbouring SPGs establish septate junctions (Fig 4C and 4C’), as reported previously [30], SPG and cortex glial cells were connected to each other through adherens junctions (Fig 4C and 4C”). Based on our light and electron microscopic observations, we concluded that the medial part of the central brain contains specialized superficial cortex glial cells that encapsulate NBs and contain the highest amount of LDs.

### Superficial cortex glial cells and SPGs express *Drosophila* fatty acid binding protein (Dfabp)

**Fig 5:**
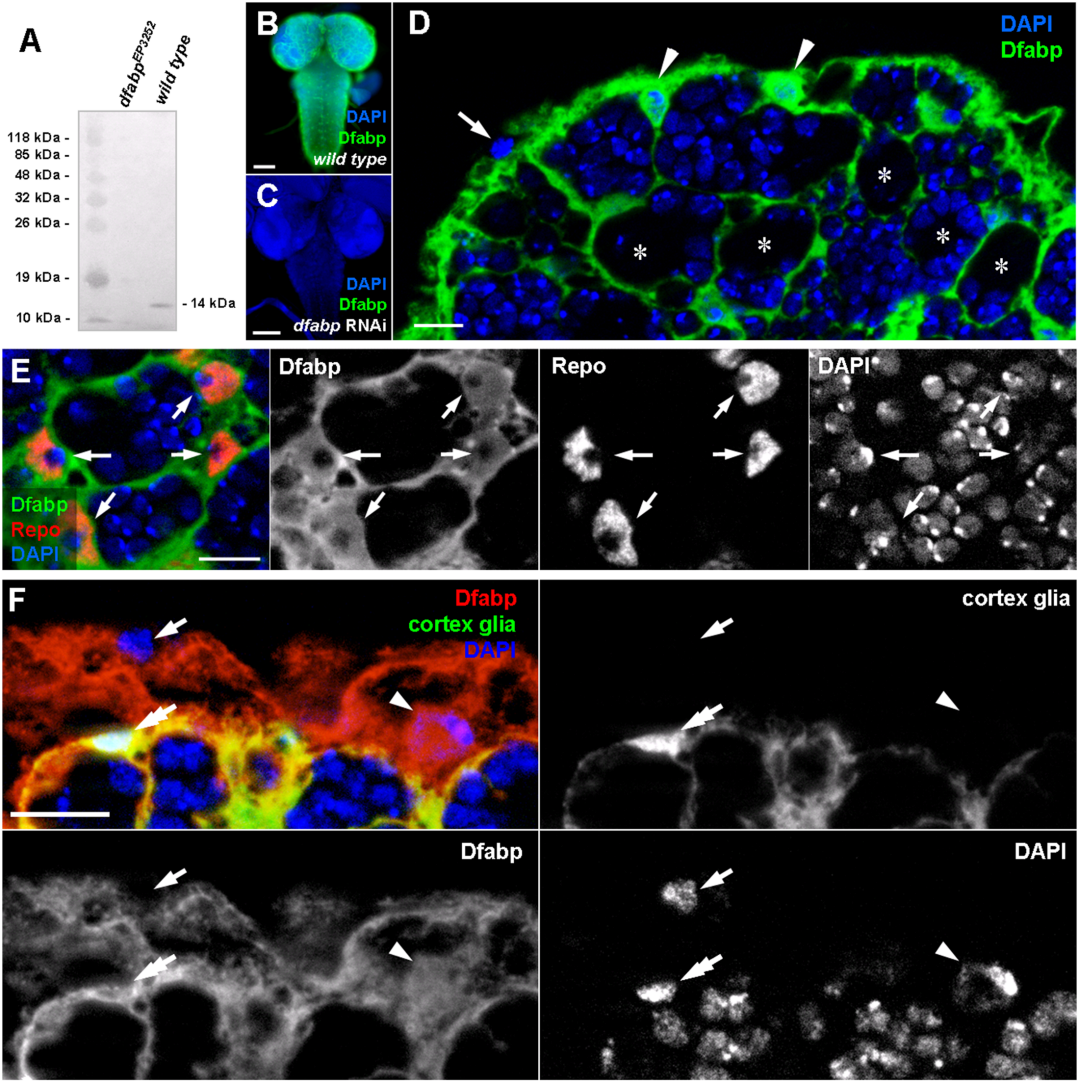
Light microscopic localisation of the Drosophila fatty acid binding protein (Dfabp) in third instar larvae. (A) Western blot performed on total protein extracts from *dfabp* ^*3252*^ homozygous mutant and wild type larvae. The Dfabp antibody labels a single band at 14kDa in the wild type sample, while no labeling is observed in samples from mutants. Immunohistochemistry on wild type (B), and on *dfabp* RNAi (C) third instar larval brains using the Dfabp antibody (green). Nuclei are stained with DAPI (blue). Note the absence of staining in RNAi silenced compared to wild type animals. (D) Higher magnification image of the dorsomedial part of the central brain. The Dfabp antibody reveals a thin network, between neuroblasts (asterisks) and their daughter cells. Two Dfabp-positive (arrowheads), and one unlabeled soma (arrow) are visible at the brain surface. (E) Double labeling against Dfabp (green) and the glial-specific protein Repo (red). Note that Dfabp is present in the cytosol and in the nucleus of glial cells (arrows). (F) Double labeling for cortex glia-GFP (green) and Dfabp (red). Perineurial cells (arrow) are double negative for Dfabp and GFP. Subperineurial cells (arrowhead) are positive for Dfabp and negative for GFP. Cortex glial cells (double arrow) are double positive for Dfabp and GFP. Scalebar: B, C:100 μm; D, E, F, F’: 10 μm.

Next, we wanted to find molecular markers that would be specific to LD accumulating superficial cortex glial cells. With this objective in mind, we performed a literature based screen for lipid metabolism-related genes expressed in the *Drosophila* CNS. We found that the *Drosophila* fatty acid binding protein (*dfabp*,*CG6783*), an orthologue of the mammalian fatty acid binding protein 7 (FABP7/brain lipid binding protein, BLBP) is preferentially expressed in glial cells of the embryonic nervous system, based on *in situ* hybridization, and microarray analysis [34–37]. We were interested in whether or not this protein is indeed expressed in third instar larval glial cells. To test this, we raised polyclonal antisera against the third exon of *dfabp*. On Western blots from wild type animals a single band (14kDa) was detected corresponding the predicted molecular mass of *dfabp*, while no labeling was observed in samples from larvae homozygous for *dfabp* ^*EP3252*^, a strong hypomorf mutation of *dfabp* (Fig 5A). Since the *dfabp* ^*EP3252*^ mutation causes early larval lethality, we validated our antibody for immunohistochemistry using transgenic flies in which *dfabp* was silenced with a microRNA based RNAi construct specifically expressed in all glial cells using repo-Gal4. The antibody strongly labelled the brains of wild type but not the *dfabp* RNAi silenced brains, which confirms the specificity of the anti-Dfabp antibody (Fig 5B and 5C).

**Fig 6:**
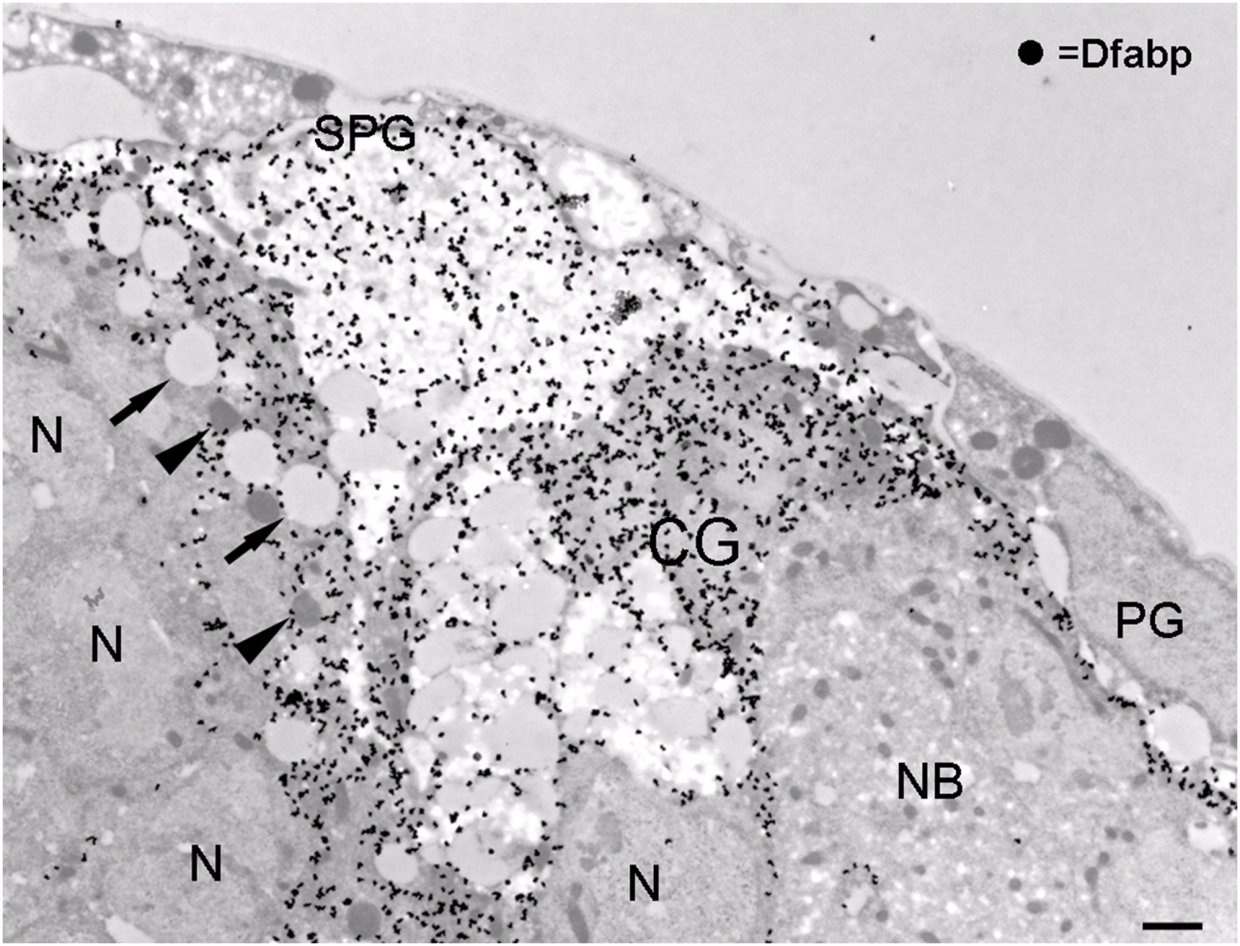
Ultrastructural localisation of Dfabp. Post-embedding silver-intensified immunogold labeling for Dfabp on freeze-substituted LR White-embedded material. An intense labeling can be seen over subperineurial (SPG) and superficial cortex (CG) glial cells. Note the absence of labeling over perineurial glia (PG), neurons (N), or neuroblasts (NB). Arrows: lipid droplets, arrowheads: mitochondria. Scalebar: 1 μm

The Dfabp antibody showed a strong staining in a layer at the surface of the brain and also a deeper thin network likely to correspond to a subset of glial cells. The pattern of the immunoreactivity seemed to surround clusters of unlabeled cell bodies (Fig 5D). In addition, some cells located at the brain surface were immunonegative for Dfabp. To confirm the presumed glial localisation of Dfabp, we performed double labeling on larval brains against Dfabp and reverse polarity (Repo), a glial-specific protein [38]. Dfabp was detected in the cytoplasm and in the nucleoplasm of the Repo-positive glial cells (Fig 5E), while no labelling was obvesrved in neurons. Cortex glial cells are known to form a network between neurons, so this cell type seemed to be a good candidate to express Dfabp. To verify this, we immunostained Dfabp and the cortex glia of a transgenic line expressing NP2222-Gal4 driven GFP [39] (Fig 5F). The GFP signal overlapped with the Dfabp labeling, but another layer above the cortex glial cells was also immunoreactive for Dfabp, possibly representing the subperineurial glial layer. Perineurial cells located at the brain surface were negative for Dfabp. We wanted to confirm our light microscopic observations at the EM level as well. Accordingly, we carried out post-embedding immunogold labeling to determine the precise cellular and subcellular localisation of Dfabp. In the EM, we found cortex and subperineurial cells to be strongly immunoreactive for Dfabp, while perineurial cells, neuroblasts, and neurons were always immunonegative (Fig 6). In glial cells, Dfabp labeling was detected over the cytosol, nucleoplasm, and glycogen areas. No labeling was observed over mitochondria, ER, or LDs. Superficial cortex glial cells, containing numerous LDs, were always strongly immunopositive for Dfabp (Fig 6). We concluded that our Dfabp antibody selectively labels the subperineurial and the lipid accumulating superficial cortical glial cells in the third instar larval brain.

## Discussion

**Fig 7:**
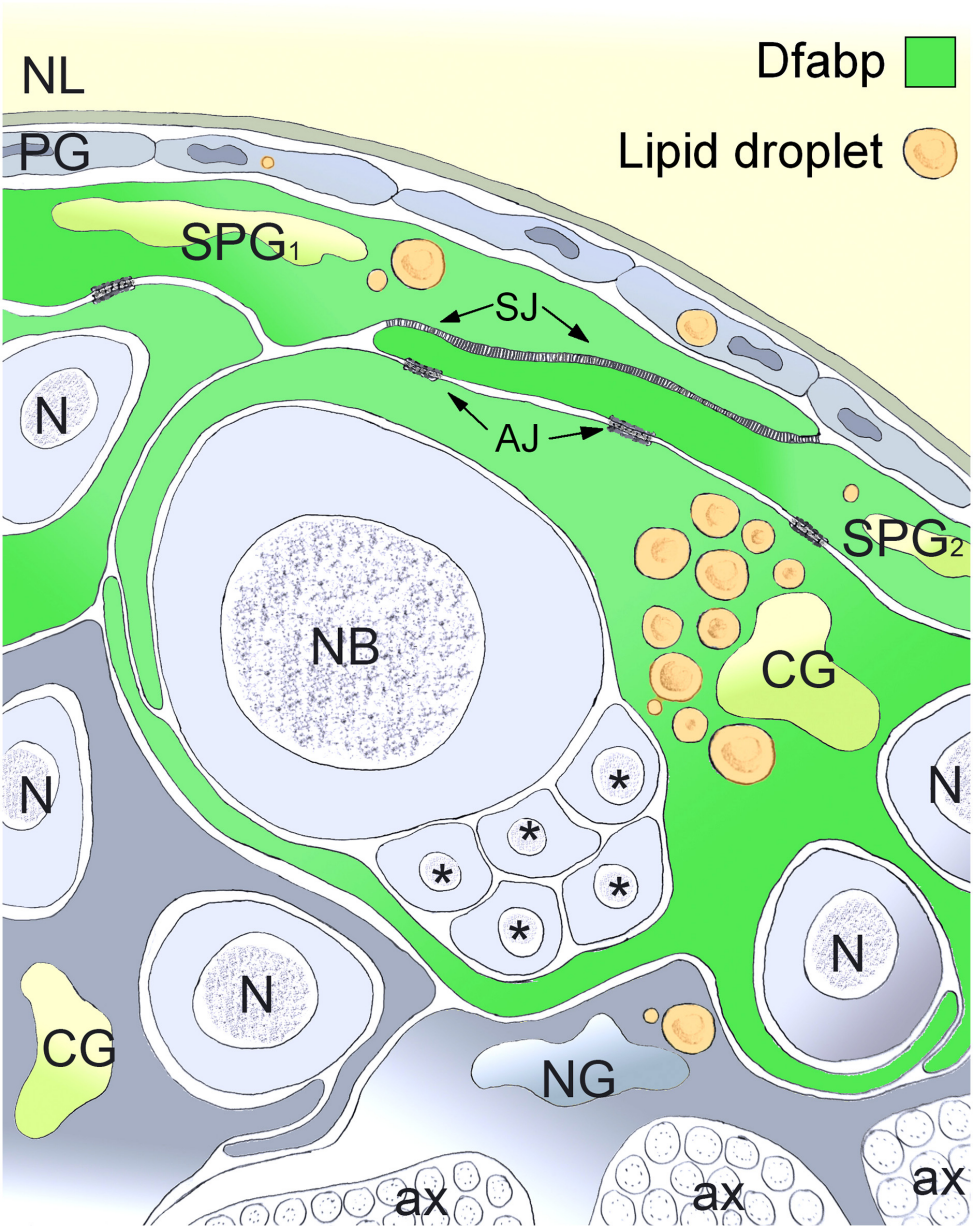
Schematic illustration of the relative distribution of lipid droplets between glial cell types and the localization of Dfabp. LDs are concentrated in large clusters in the perinuclear region of glial cells but are not present at all in neurons (N). Specialized superficial cortex glial cells (CG) insulating neuroblasts (NB) and their daughter cells (asterisks) accumulating the highest amount of LDs. Neighboring subperineurial cells (SPG) establish septate junctions (SJ), while SPG and superficial cortex glial cells are connected to each other through adherens junctions (AJ). The Drosophila fatty acid binding protein (Dfabp) is expressed in LD accumulating superficial cortex glial cells and subperineurial (SPG) cells, and is localized in the cytosol and in the nucleus. NL: neural lamella, PG: perineural glia, NG: neuropil glia, ax: axon.

LDs are common organelles of eukaryotic cells, participating in a variety of cellular processes. Although lipids are highly enriched in the nervous system, the spatio-temporal distribution and physiological function of LDs in the developing brain is poorly understood. In this work, we demonstrated the highly specific spatio-temporal distribution of LDs in the nervous system of Drosophila. Key findings of our work are summarized in Fig 7.

### Lipid droplets in glial cells

We showed that LDs are present throughout the entire larval brain but are preferentially enriched in the medial part of the central body. LDs are concentrated in large clusters in the perinuclear region of glial cells but are not present at all in neurons. In this brain area the majority of LDs is found in the closest vicinity of neuroblasts. We found that LDs are absent in the embryonic stage, and in the first larval instars but the density of LDs gradually increases from the beginning of the second larval stage until the end of the wandering period of the last larval stage, where it reaches its highest value, then it decreases gradually till the end of the pupal stage. Virtually no LD is found in the brain of a pharate adult fly. This dataset suggests that the brain’s LD pool is not constant but changes dynamically in parallel with the developmental changes during metamorphosis. The unique spatial segregation of LDs between neurons and glial cells, and the time-course changes in the amount of LDs together suggest that LDs may serve specific functions during brain development through a novel, yet unknown way of neuron-glia interaction. The amount of LDs start to decrease in the middle of the pupal stage, just after the mitotic activity of neuroblasts terminates and newborn neurons start to develop processes [40,41]. The dynamics of LDs raise the possibility that during development, free fatty acids mobilised from LDs may promote distinct cellular processes, such as cell division and cell membrane expansion, two important features of the developing brain [42,43]. The development of neural processes of newborn neurons requires a huge amount of lipids, which can only be provided from the accumulated LDs, since the animal is incapable of feeding inside the puparium. Another explanation for the function of LDs could be that they provide energy during brain development. However, given the amount of glycogen accumulated by neurons and glia, it is unlikely that LDs would function as energy sources (S3 Fig). In rats it has been shown that the brain does not use fatty acids for energy production [44]. At the same time, membrane building blocks can only be synthesized from lipids mobilised from the LD pool and not from the glycogen stores. [45] The fact that Drosophila is sterol auxotroph [46] and cannot synthesize polyunsaturated fatty acids, both of which are essential for brain development [47], makes LDs the only depots where these lipids can be stored until processing.

### Superficial cortex glial cells accumulate lipid droplets

We further showed that LDs are not distributed equally between various glial cell types, with specialized superficial cortex glial cells insulating neuroblasts accumulating the highest amount of LDs. In addition, we found in the EM that neighboring SPGs establish septate junctions [30], while SPG and superficial cortex glial cells are connected to each other through adherens junctions. This cell type-specific segregation of cell adhesion structures might promote the assembly and maintenance of a highly organized glial scaffold during development. Taken together, these data suggest that perineurial, subperineurial and superficial cortex glial cells are anatomically and functionally related components of the nervous system.

### Superficial cortex glial cells and subperineurial cells express Dfabp

In addition, we found that the *Drosophila* fatty acid binding protein *(*Dfabp, *CG6783)* is expressed in superficial cortex glial cells and subperineurial (SPG) cells, and is localized in the cytosol and in the nucleus. Nuclear localisation of the fatty acid binding proteins was reported previously in mammals [48,49]. Since cytosolic fatty acid binding proteins are known to be cellular lipid carriers [50], the presence of Dfabp in SPGs and superficial cortex glial cells raises the possibility that Dfabp transfer fatty acids from the hemolymph into glial LDs. The mammalian orthologue of dfabp, the brain lipid binding protein (BLBP/FABP7) is expressed in radial glial cells in the developing brain and in astrocytes in the mature nervous system [48,51,52]. FABP7 KO mice have a decreased number of astrocytes, neural stem cells and early progenitor cells in the developing brain [53]. Interestingly, FABP7 mice also exhibit increased fear memory and enhanced anxiety without any visible histological abnormalities [54]. It should be noted that investigating the function of FABPs in mammals is problematic, because three fatty acid binding proteins (FABP3, FABP5, FABP7) are present in the mammalian nervous system with partially overlapping expression pattern [49], and in KO animals other FABPs may compensate for the loss of the gene of interest. For that reason, Drosophila offers an excellent opportunity to study the function of *dfabp*, since there is no other gene in the genome that can compensate for the loss of *dfabp*. The physiological function of LDs was investigated in the *Drosophila* ring gland [55] and in the adult retina [56] but to the best of our knowledge this is the first study that presents detailed anatomical data about LDs in the *Drosophila* nervous system. We showed that LDs are transient organelles of *Drosophila* glial cells and the majority of these LDs are localised in specialized superficial cortex glial cells that express the Drosophila fatty acid binding protein Dfabp.

## Supporting Information

S1 Fig (A) Representative EM images from the medial part of the central brain of third instar larvae where glial membranes were stained with repo-Gal4 driven membrane-targeted HRP. Glial membranes (arrows) are darkly stained whereas neuronal membranes are fainter (double arrow). Note the accumulation of LDs (asterisks) in glial cells (G). HRP accumulates in endosomal structures (arrowheads) as well. Glial membranes (arrows) are darkly stained whereas neuronal membranes are fainter (double arrow). Scalebar: 5 μm (A), 2 μm (B).
(TIF)

S2 Fig Representative toluidine blue stained resin section from the medial part of the central brain. Note that superficial cortex glial cells (double arrows) accumulates a high amount of LDs compared to cortex glial cells located deeper in the brain (arrows). Asterisks: neuroblasts. Scalebar: 2 μm.
(TIF)

S3 Fig Representative EM image from the medial part of the central brain of third instar larva which was stained with potassium ferrocyanide-reduced osmium. In this specimen glycogen particles are darkly stained and their rosette structure is clearly visible. G: glial cell, asterisks: lipid droplet. Scalebar: 1 μm.
(TIF)
