# Supplementary figures and images for: Specialized Cortex Glial Cells Accumulate Lipid Droplets in Drosophila melanogaster
Source: PLoS One. 2015 Jul 6;10(7):e0131250. doi: 10.1371/journal.pone.0131250 (PMC4493057; doi:10.1371/journal.pone.0131250)

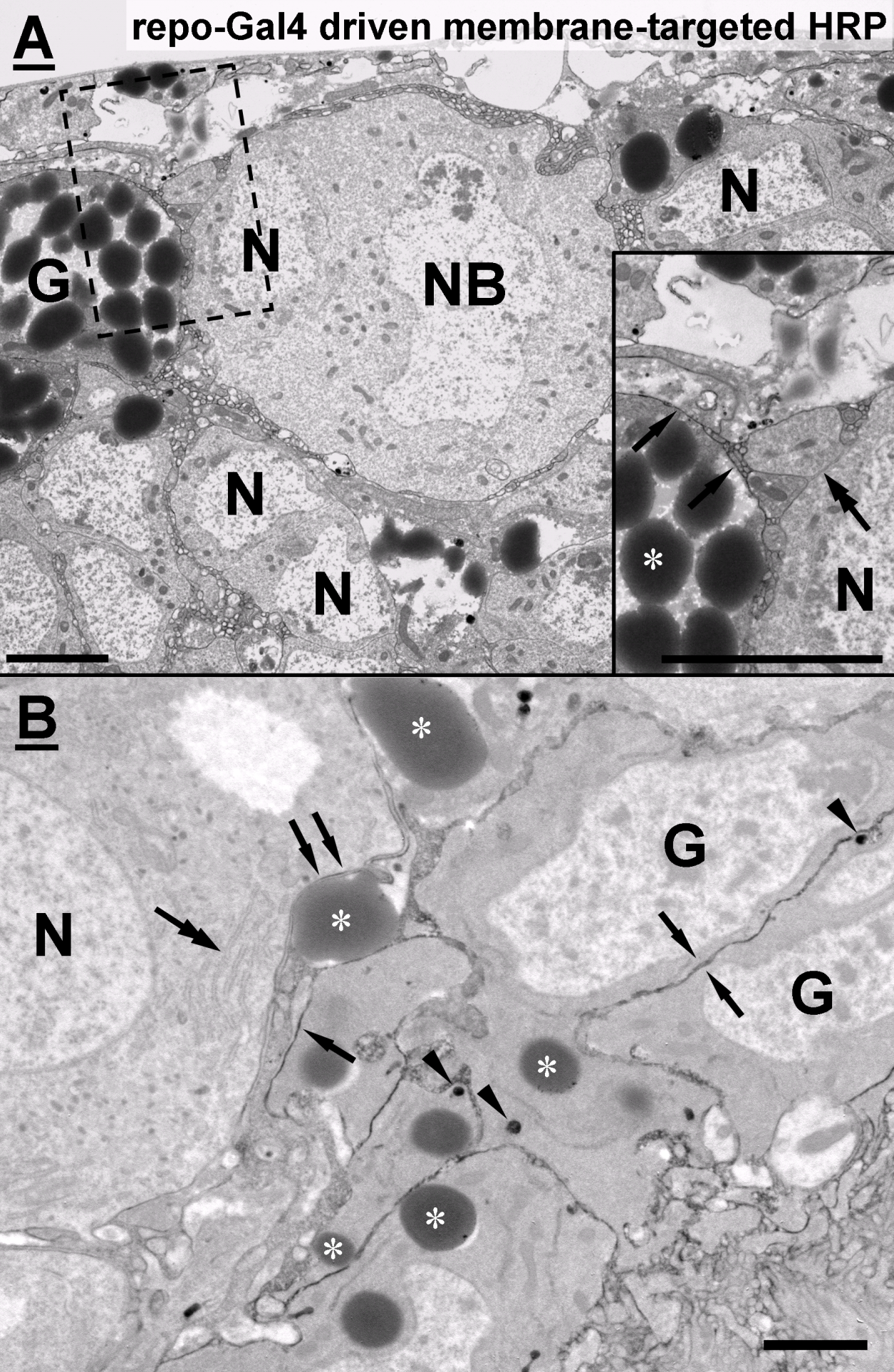

Supplement: S1 Fig — Glial membranes (arrows) are darkly stained whereas neuronal membranes are fainter (double arrow). Note the accumulation of LDs (asterisks) in glial cells (G). HRP accumulates in endosomal structures (arrowheads) as well. Glial membranes (arrows) are darkly stained whereas neuronal membranes are fainter (double arrow). Scalebar: 5 μm (A), 2 μm (B). (TIF) [file pone.0131250.s001.TIF]

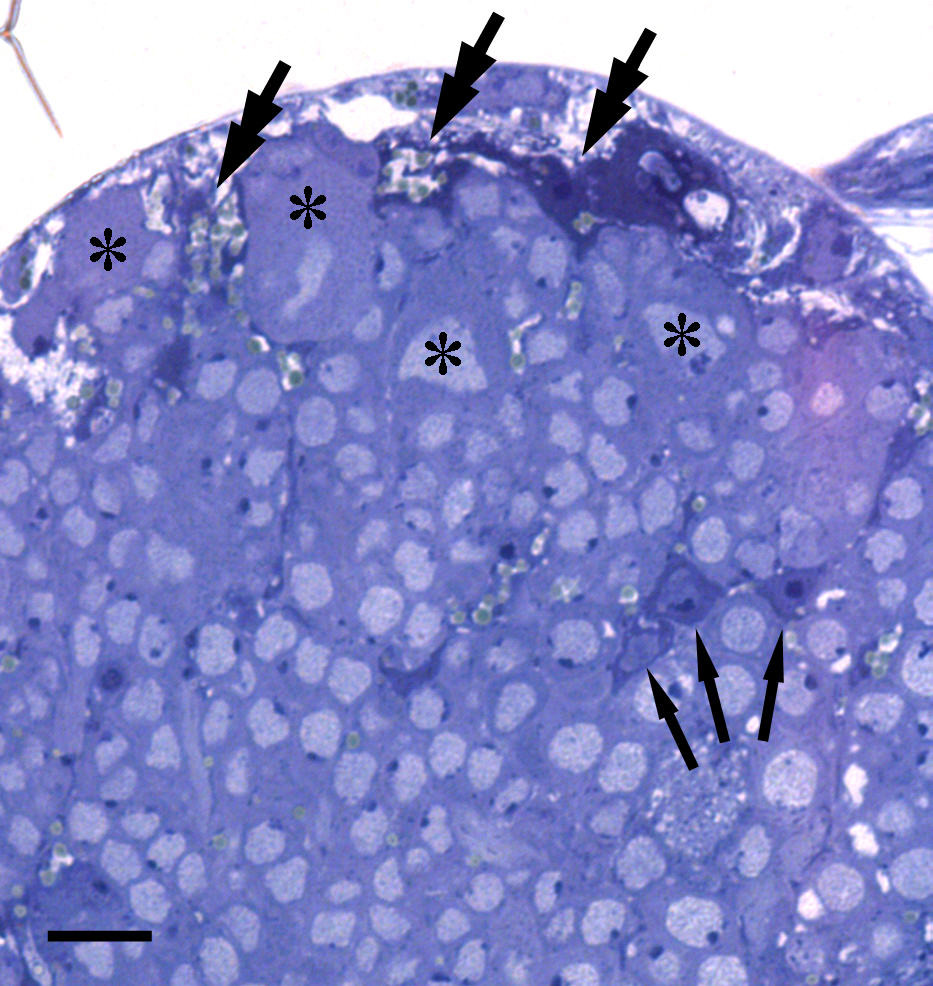

Supplement: S2 Fig — Note that superficial cortex glial cells (double arrows) accumulates a high amount of LDs compared to cortex glial cells located deeper in the brain (arrows). Asterisks: neuroblasts. Scalebar: 2 μm. (TIF) [file pone.0131250.s002.TIF]

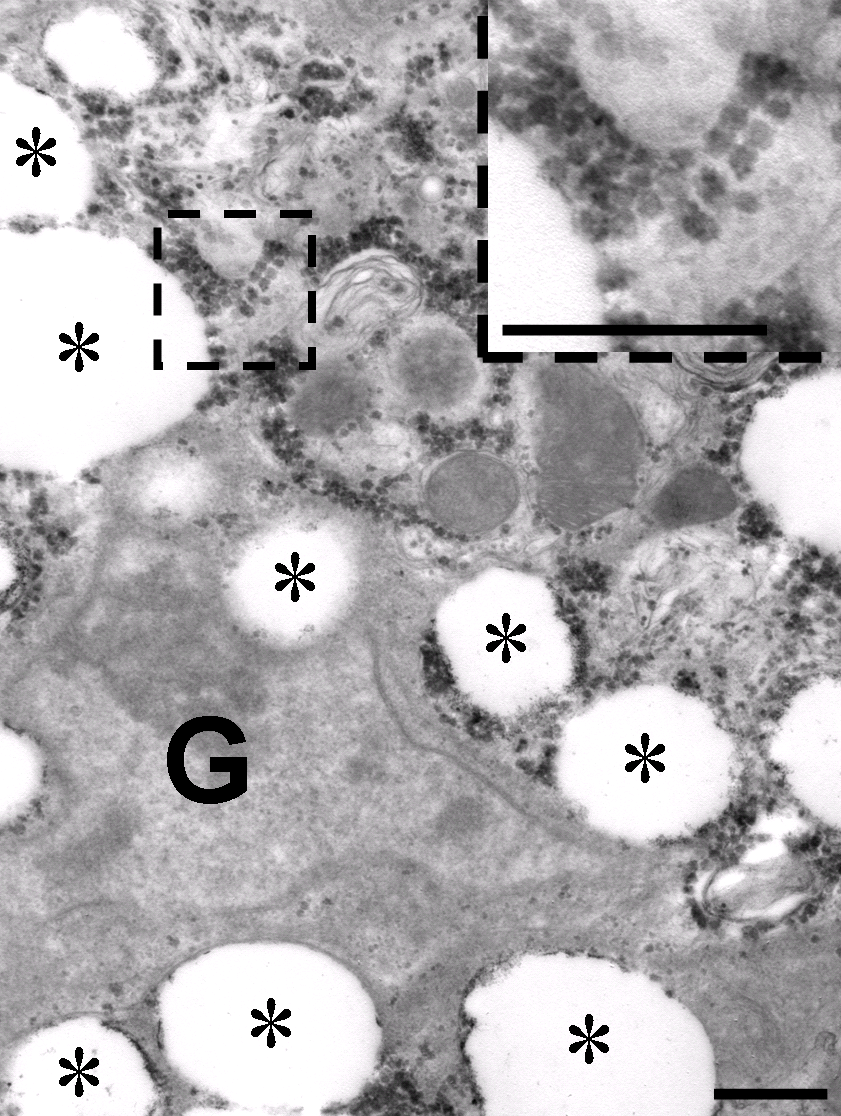

Supplement: S3 Fig — In this specimen glycogen particles are darkly stained and their rosette structure is clearly visible. G: glial cell, asterisks: lipid droplet. Scalebar: 1 μm. (TIF) [file pone.0131250.s003.tif]
